# MiR‐223‐3p affects the proliferation and apoptosis of HCAECs in Kawasaki disease by regulating the expression of FOXP3

**DOI:** 10.1002/iid3.939

**Published:** 2023-07-27

**Authors:** Ronghao Zheng, Jing Xie, Weijie Li, Jianping Shang, Zuliang Shi, Songbai Zhu, Lin Gui, Li Huang, Lan Shu, Donglei Liu, Yi Gong, Xiaohui Li, Wanxia Chai, Xiaofen Huang, Xiaolin Wu, Jing Yue

**Affiliations:** ^1^ Department of Pediatric Nephrology, Rheumatology, and Immunology, Maternal and Child Health Hospital of Hubei Province, Tongji Medical College Huazhong University of Science and Technology Wuhan China; ^2^ Department of Pediatric Nephrology, Rheumatology, and Immunology, Maternal and Child Health Hospital of Hubei Province Hubei University of Medicine Shiyan Hubei China; ^3^ Department of Clinical Laboratory, Maternal and Child Health Hospital of Hubei Province, Tongji Medical College Huazhong University of Science and Technology Wuhan China; ^4^ Emergency Department, Maternal and Child Health Hospital of Hubei Province, Tongji Medical College Huazhong University of Science and Technology Wuhan Hubei Province China

**Keywords:** apoptosis, FOXP3, inflammation levels, Kawasaki disease, miR‐223‐3p

## Abstract

**Objective:**

Kawasaki disease (KD) can lead to permanent damage to coronary structures, the pathogenesis of which remains unknown. This experiment was designed to investigate whether miR‐223‐3p secreted in the serum of KD patients affects the proliferation and apoptosis of HCAECs in KD by regulating FOXP3.

**Methods:**

Blood samples were collected in acute febrile phase of KD, after IVIG treatment, and from healthy controls. Transfected into HCAECs cells by synthetic FOXP3 siRNA/NC. A co‐culture system was established between HCAECs cells transfected with FOXP3 siRNA/NC and THP1 cells added with three sera.

**Results:**

Compared with the control group, the expressions of miR‐223‐3p, RORγt, and Th17 in serum of KD patients were significantly upregulated, and the expressions of TGF‐β1, FOXP3 and Treg were significantly downregulated. At the same time, the levels of IL‐6, IL‐17, and IL‐23 were significantly increased, and the levels of IL‐10 and FOXP3 were significantly decreased. After IVIG treatment, the patient's above results were reversed. The serum of KD patients increased the expression of miR‐223‐3p and inhibited the expression of FOXP3 in HCAECs cells. IVIG serum is the opposite. Overexpression of miR‐223‐3p also promoted the apoptosis of HCAECs. In addition, serum from KD patients promoted apoptosis, whereas serum after IVIG treatment inhibited apoptosis. KD patient serum downregulated the expression of FOXP3, Bcl2, TGF‐β1 and IL‐10 in cells, and upregulated the expression of caspase3, Bax, IL‐17, IL‐6, and IL‐23. The opposite results were obtained with IVIG‐treated sera.

**Conclusion:**

miR‐223‐3p secreted in serum of KD patients can regulate the expression of FOXP3 and affect the proliferation, apoptosis, and inflammation of cells.

## Graphical abstract

It was verified that Kawasaki disease (KD) serum has high level of miR‐223‐3p and low level of FOXP3. A co‐culture system was established with HCAECs cells and THP1 cells treated with three serum samples. It was verified that serum miR‐223‐3p in KD patients may affect cell proliferation, apoptosis, the expression of inflammatory cytokines, and the level of angiogenesis factors by regulating FOXP3.

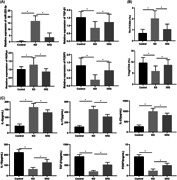

## 1 INTRODUCTION

Vascular injury is the leading cause of morbidity and mortality in Kawasaki disease (KD),^1^, ^2^ also a major reason for heart disease in children worldwide.^3^ Diagnosis is mainly based on the clinical features including fever lasting 5 days or more with the presence of bilateral conjunctival injections, oral changes, cervical lymphadenopathy, extremity changes, and a pleomorphic rash.^4^ KD can be treated with intravenous immunoglobulin (IVIG), oral aspirin, or anti‐inflammatory agents such as TNF‐α blockers and IL‐1 receptor antagonist.^5^, ^6^, ^7^ Particularly, early and proper use of IVIG is very important for preventing cardiovascular complications.^8^

miRNAs can participate in various biological processes such as cell apoptosis, angiogenesis, and differentiation.^9^, ^10^ miR‐223 is a blood‐forming lineage miRNA that is exported in cells such as blood platelets and leukocytes of the bone marrow.^11^ Chen et al.^12^ confirmed the upregulation of hsa‐miR‐223‐3p in the plasma of acute KD patients by microarray gene chip, and speculated that it is involved in the pathogenesis of KD. Clinical studies have shown that in the diseased state of KD, blood cells are excited and release more miRNAs into the serum, leading to a rise in the levels of miRNAs, such as miR‐223, in vascular wall ECs and vascular smooth muscle cells (VSMCs). Increased miR‐223 in vascular cells from KD patients increases vascular cell damage, ultimately leading to vascular damage, such as vascular thrombosis and aneurysm.^13^ Therefore, miR‐223‐3p is an essential regulator of intracellular damage in KD.

Imbalance of Th17/Th1 and Th2/Treg affects the progression of KD, with increased Th17/Th1 responses, increased levels of IL‐6, IL‐10, IL‐17A, IFN‐γ, and IP‐10, decreased Th2/Treg responses, and decreased IL‐6, IL‐10, IL‐17A, IFN‐γ, and IP‐10 levels ‐4. Decreased expression of IL‐5, FoxP3, and TGF‐β.^14^ Forkhead Box P3 (FOXP3) is mainly expressed in the T regulatory cell (Treg) subpopulation in CD4+ cells, and is the most important marker of this cell subtype and is involved in the activation of Treg cells.^15^ Whether miR‐223 secreted in serum of KD patients can regulate the apoptosis of coronary endothelial cells by regulating FOXP3, and then participate in the progression of KD, has not been reported yet. Therefore, this experiment was to assess the effect of FOXP3 interference on HCAECs cells by collecting blood samples from the acute phase of KD, after IVIG treatment and healthy controls. To evaluate the regulatory effect of serum miR‐223‐3p in KD patients on FOXP3 in HCAECs cells, and its effects on cell apoptosis, inflammasome, inflammatory cytokine expression, and angiogenesis factors.

## 2 MATERIALS AND METHODS

### 2.1 Main reagent

Trizol (15596026; Ambion). All ELISA kits were purchased from Bioswamp. All antibodies were from bioswamp. Opti‐MEM (31985‐062; Gibco). Lipofectamine® RNAiMAX (13778030; Invitrogen). MTT (M1025; Solarbio). DMSO (D2650; SIGMA). Annexin V‐FITC/PI Apoptosis Detection Kit (556547; BD).

### 2.2 Clinical sample testing

From August 2021 to October 2022, 15 children with KD were randomly selected as the KD group (age: 33.3 ± 24.6 months), 15 healthy children were randomly selected as the control group (age: 38.5 ± 38.4 months), children in the IVIG group were KD children after IVIG treatment. Collect serum from children in the acute phase of KD, post‐IVIG KD serum (2 g/kg IVIG treatment) and health serum (similar age, healthy, without any infection, or inflammation). Blood collection by vein.

### 2.3 Cell culture

HCAECs cells were derived from Shanghai Saibaikang. Thp‐1 cells were obtained from the Shanghai Cell Bank of the Chinese Academy of Sciences. Move the cells to a 37°C water bath. After completely thawing, pipette the cell suspension into a centrifuge tube, add 4 mL of complete medium, then centrifugation is performed. Incubate in a 37°C, 5% CO_2_ incubator. Divide cells into four groups: Normal group: HCAECs cells, cultured normally; Control group: HCAECs cells + health serum; KD group: HCAECs cells + KD serum; IVIG group: HCAECs cells + post‐IVIG KD serum.

### 2.4 qRT‐PCR

Take the cell samples into a 1 mL Trizol homogenization tube for total RNA extraction. RNA is then reverse‐transcribed into cDNA. PCR amplification of the prepared cDNA. The reaction program was: predenaturation at 95°C for 3 min; 95°C for 5 s, 56°C for 10 s, and 72°C for 25 s (40 cycles). The relative content of mRNA was calculated using $2^{–\Delta \Delta_{t}}$. PCR primers are shown in Table 1.

**Table 1:** Primer sequence.

Primer name	Sequence	Size (bp)
TGF‐β1‐F	TGTGGCTACTGGTGCTGAC	275
TGF‐β1‐R	CTCCTTGGCGTAGTAGTCG	
RORγt‐F	GCTGACCCCTGACCGAT	184
RORγt‐R	TGTCTCCCTGTAGGACTTGC	
FOXP3‐F	GCTGGCAAATGGTGTCTG	258
FOXP3‐R	GAGCCCTTGTCGGATGAT	
miR‐223‐3p‐F	GGGGTGTCAGTTTGTCAAAT	57
miR‐223‐3p‐R	CTGGTGTCGTGGAGTCGG	
GAPDH‐F	GGGAAACTGTGGCGTGAT	299
GAPDH‐R	GAGTGGGTGTCGCTGTTGA	
U6‐F	CTCGCTTCGGCAGCACA	94
U6‐R	AACGCTTCACGAATTTGCGT	

### 2.5 ELISA

ELISA kits were used to detect the expressions of IL‐6, IL‐17, IL‐23, IL‐10, TGF‐β1, and FOXP3. The procedure was performed in accordance with the instructions of the kit.

### 2.6 Synthesis and transfection of miR‐223 mimics/NC and FOXP3 siRNA

Mimics/NCs were synthesized according to the sequence of hsa‐miR‐223‐3p (UGUCAGUUUGUCAAAUACCCCA), and transfected into cultured HCAECs cells. siRNA/NC was synthesized according to the sequence of FOXP3 and transfected into HCAECs cells by lipofection. Before transfection, and the cell confluency was 90% at the time of transfection. Dilute 5 µL Lipofectamine® RNAiMAX in 250 µL Opti‐MEM. The two were mixed and incubated at room temperature for 20 min. Place the cell plate in a 37°C incubator, change to fresh medium 4 h after transfection, and incubate for 48 h. Finally, the expression of the transgene was detected.

### 2.7 Cell co‐culture

Establish a cell co‐culture system. Divide the cells into nine groups: Control group (lower chamber: HCAECs cells, upper chamber: THP1 cells + healthy serum), siRNA group (lower chamber: HCAECs cells + siRNA, upper chamber: THP1 cells + healthy serum), siRNA‐NC group (lower chamber: HCAECs cells + siRNA‐NC, upper chamber: THP1 cells + healthy serum), KD group (lower chamber: HCAECs cells, upper chamber: THP1 cells + KD serum), KD + siRNA group (lower Chamber: HCAECs cells + siRNA, upper chamber: THP1 cells + KD serum), KD + siRNA‐NC group (lower chamber: HCAECs cells + siRNA‐NC, upper chamber: THP1 cells + KD serum), IVIG group (lower chamber: HCAECs cells, upper chamber: THP1 cells + IVIG serum), IVIG + siRNA group (lower chamber: HCAECs cells + siRNA, upper chamber: THP1 cells + IVIG serum), IVIG + siRNA‐NC group (lower chamber: HCAECs cells + siRNA‐NC, upper compartment: THP1 cells + IVIG serum). The coronary artery endothelial cells were collected, and the concentration of the cell suspension was adjusted with complete medium. Add transfected/untransfected FOXP3 siRNA and NC coronary artery endothelial cells in the lower chamber of the 6‐well plate Transwell, 2.6 mL/well. Collect THP1 cells, adjust the concentration of cell suspension with basal medium (without serum), add THP1 cells to the upper chamber, 1.2 mL/well. Afterwards, 0.3 mL of KD serum, IVIG treatment serum and healthy serum were added to the upper chamber, respectively. After 24 h of treatment, follow‐up testing.

### 2.8 MTT

Take out the cell culture plate, add 10 μL MTT solution to each well, and culture for 4 h. Add 150 uL DMSO solution. The absorbance of each well was measured at 490 nm.

### 2.9 Flow cytometry

Take 1 × 10^6^ cells resuspended in medium, and centrifuge. Add Annexin V‐FITC and PI, and incubate at 4°C for 30 min. Three‐hundred microliters of PBS was added and flow cytometry was performed immediately. Analysis was performed using NovoExpress analysis software.

### 2.10 Western blot

Add 200 μL of lysate per 1 × 10^6^ cells. Select stacking gel at 80 V for 40 min, separating gel at 120 V for 50 min, constant pressure 90 V for 50 min, and 5% nonfat milk powder at room temperature and block overnight at 4°C. Add primary antibodies (FOXP3 1:1000, caspase3 1:1000, Bcl2 1:1000, Bax: 1000, GAPDH 1:1000), and incubated for 1 h. The secondary antibody Goat anti‐Rabbit IgG (1:20000) was incubated at room temperature for 1 h. After adding ECL luminescent solution, color development was performed and the grayscale values of the relevant bands were read, and each group was repeated three times.

### 2.11 Data analysis

The data measured are expressed as mean ± standard deviation. One‐way ANOVA was used for comparison among multiple groups, and LSD test was used for pairwise comparison of means. Statistical analysis was performed using SPSS 17.0 software. The difference was statistically significant at *p* < .05.

## 3 RESULTS

### 3.1 Expression changes of miR‐223‐3p and FOXP3 in patients with Kawasaki disease

**Figure 1:**
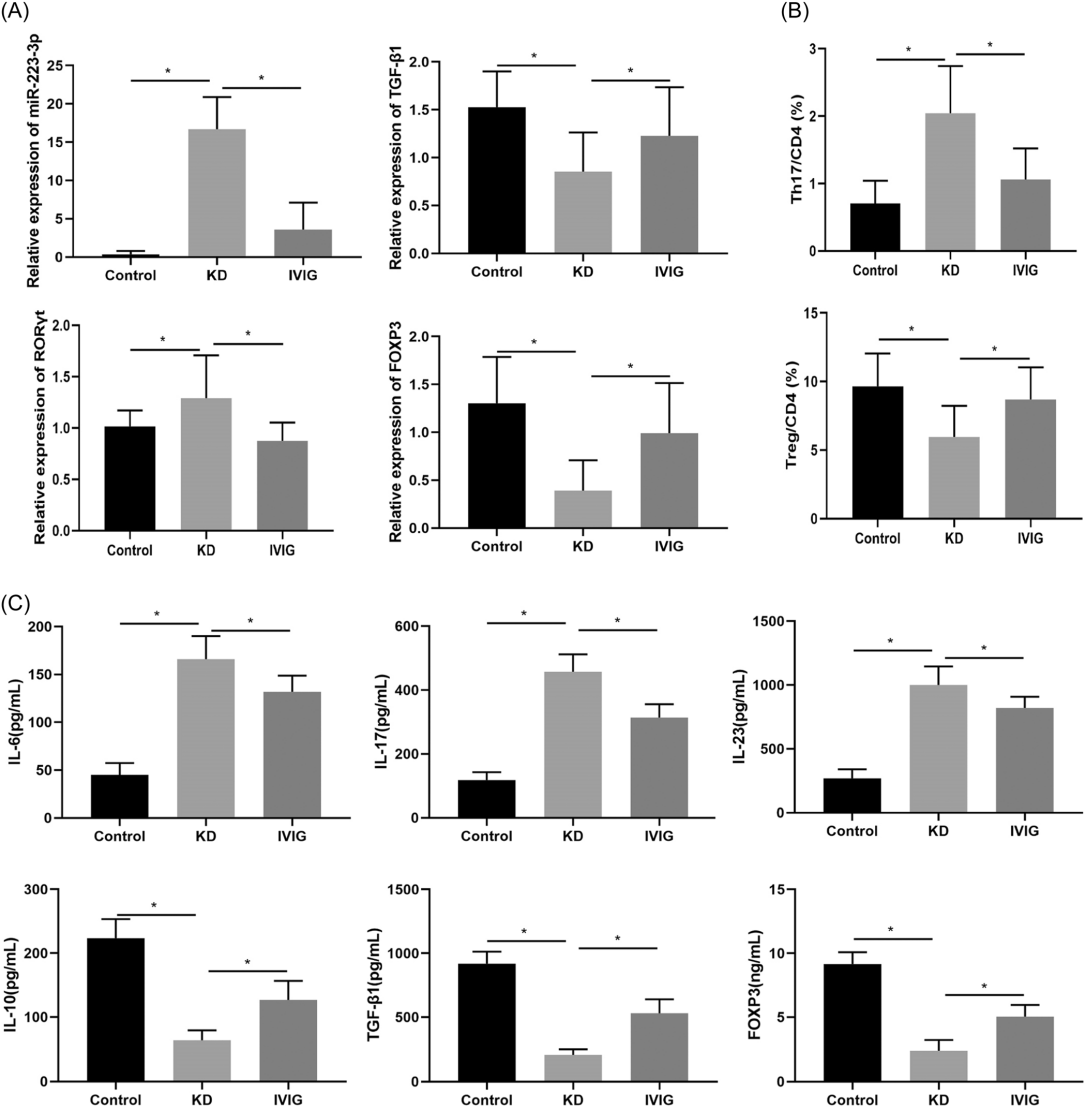
Expression changes of miR‐223‐3p and FOXP3 in patients with Kawasaki disease. (A) qRT‐PCR detection of serum miR‐223‐3p, TGF‐β1, RORγt, and FOXP3 expression; (B) Flow cytometry detection of serum Th17 and Treg cells. (C) The expressions of IL‐6, IL‐17, IL‐23, IL‐10, TGF‐β1, and FOXP3 in serum were detected by ELISA.

By collecting clinical samples, we found that compared with the control group, the expression of miR‐223‐3p and RORγt in the serum of acute KD patients was significantly upregulated, and the expressions of TGF‐β1 and FOXP3 were significantly downregulated. Compared with the KD group, the expression of miR‐223‐3p and RORγt in the serum of patients after IVIG treatment was significantly downregulated, and the expressions of TGF‐β1 and FOXP3 were significantly upregulated, Figure 1A. Compared with the control group, the proportion of Th17 cells in KD patients was significantly increased, the proportion of Treg cells was significantly decreased. The proportion of Th17 cells was significantly decreased and the proportion of Treg cells was significantly increased after IVIG treatment (Figure 1B). ELISA showed that (Figure 1C), the levels of IL‐6, IL‐17, and IL‐23 in the KD group were significantly higher than those in the control group, and the levels of IL‐10, TGF‐β1, and FOXP3 were significantly decreased. Compared with the KD group, the levels of IL‐6, IL‐17, and IL‐23 in the IVIG group were significantly decreased, and the levels of IL‐10, TGF‐β1, and FOXP3 were significantly increased. This result indicates that the serum of KD patients has high expression of miR‐223‐3p and low expression of FOXP3. However, after IVIG treatment, the expression of miR‐223‐3p was decreased, and the FOXP3 was increased.

### 3.2 Effects of miR‐223‐3p expression on cell proliferation

**Figure 2:**
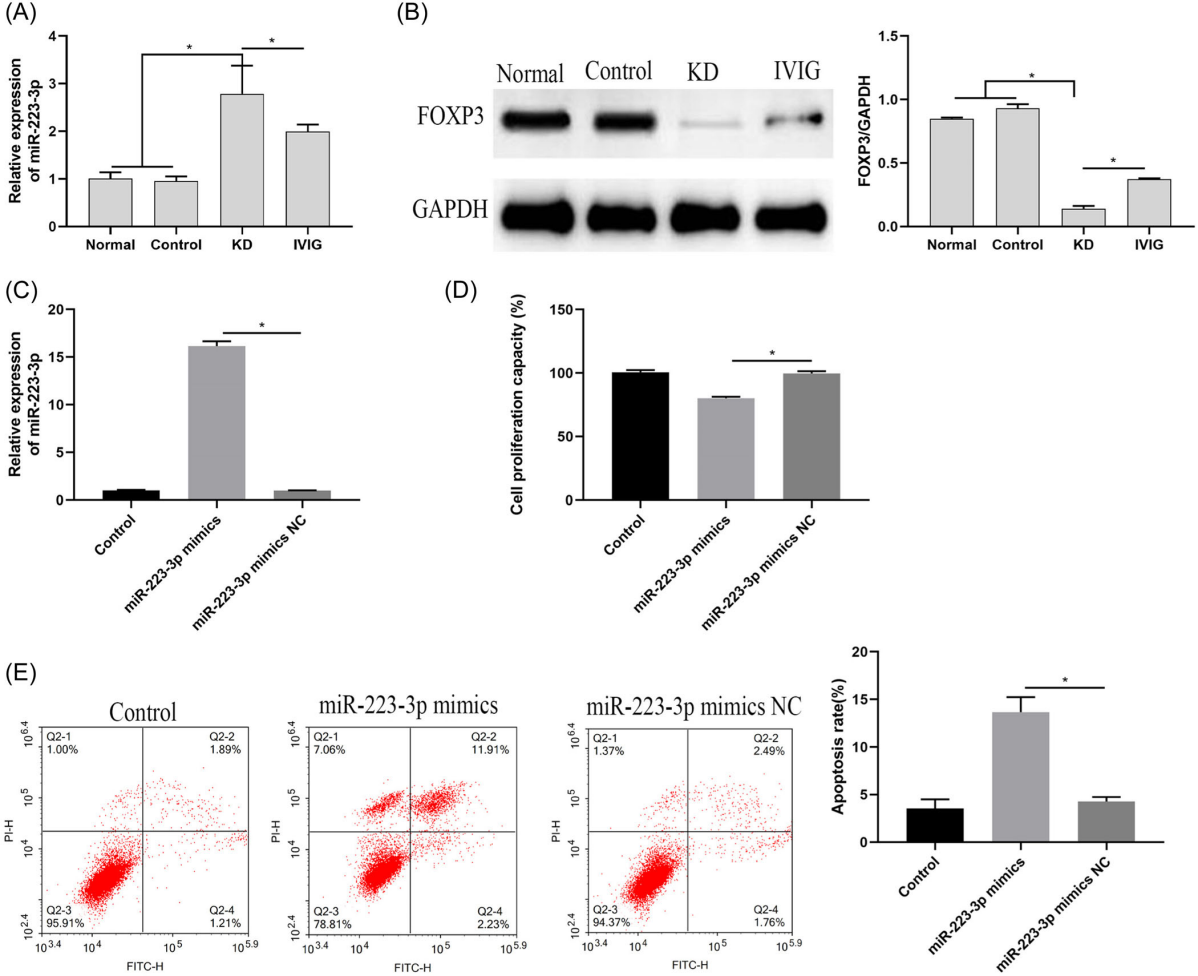
The effect of miR‐223‐3p expression on cell proliferation. (A) The expression of miR‐223‐3p was detected by qRT‐PCR; (B) The protein expression of FOXP3 was detected by western‐blot; (C) The expression of miR‐223‐3p was detected by qRT‐PCR; (D) MTT method was detected Cell proliferation; (E) Flow cytometry cell apoptosis rate.

Compared with the Normal group and the control group, the expression of miR‐223‐3p in the KD group was significantly increased, and the expression of FOXP3 was significantly decreased (Figure 2A,B). It is suggested that miR‐223‐3p may promote the progression of KD. Therefore, we further verified by constructing miR‐223 mimics/NC vector. The results showed that (Figure 2C–E), compared with the miR‐223‐3p NC group, the miR‐223‐3p mimics group increased the expression of miR‐223‐3p, inhibited cell proliferation, and promoted apoptosis. This result indicated that miR‐223‐3p could inhibit the activity of HCAECs cells.

### 3.3 The effect of FOXP3 interference on cell proliferation

**Figure 3:**
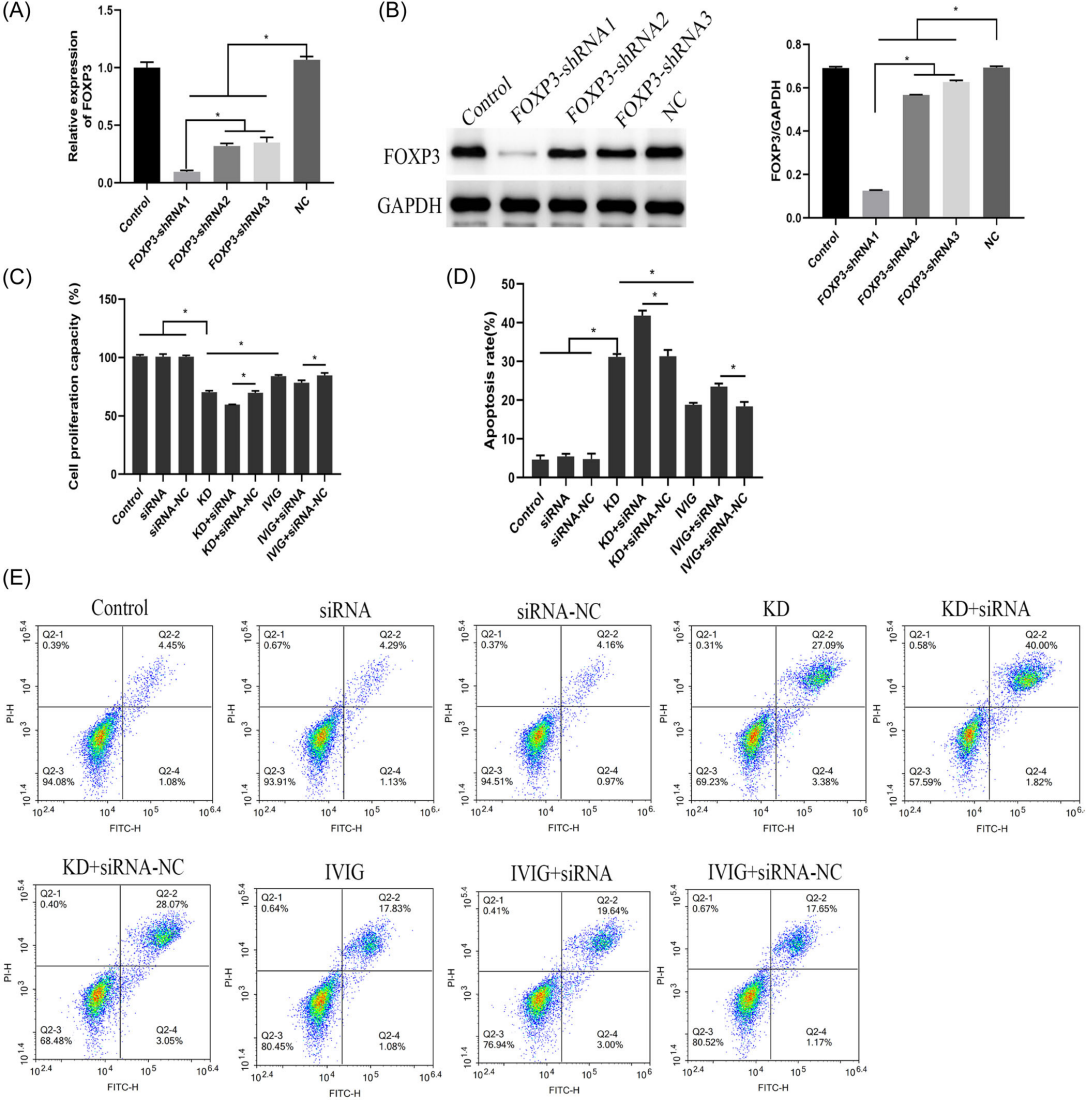
The effect of FOXP3 interference on cell proliferation. (A) qRT‐PCR detection of FOXP3 miRNA expression; (B) western‐blot detection of FOXP3 protein expression; (C) MTT detection of cell proliferation in each group; (D and E) Flow cytometry detection of cell apoptosis rate.

As shown in Figure 3A,B, compared with the NC group, the miRNA and protein expressions of FOXP3 in the cells of the FOXP3‐shRNA1, FOXP3‐shRNA2, and FOXP3‐shRNA3 groups were significantly decreased. We selected the most interfering FOXP3‐shRNA1 for subsequent experiments. Compared with the control group, the siRNA group and the siRNA‐NC group, the proliferation ability of the KD group was significantly decreased, and the apoptosis was significantly increased. Compared with the KD + siRNA‐NC group, the cell proliferation ability of the KD + siRNA group was significantly decreased, and the apoptosis rate was significantly increased. Compared with the KD group, the proliferation ability of the cells in the IVIG group was significantly increased, and the apoptosis rate was significantly decreased. At the same time, compared with the IVIG + siRNA‐NC group, the proliferation ability of the cells in the IVIG+siRNA group was significantly decreased, and the apoptosis rate was significantly increased (Figure 3C–E). This result indicated that KD serum could inhibit the activity of HCAECs cells, and this situation was more obvious after interfering with the expression of FOXP3. The serum after IVIG treatment can increase cell viability.

### 3.4 The effect of FOXP3 interference on the level of inflammation in cells

**Figure 4:**
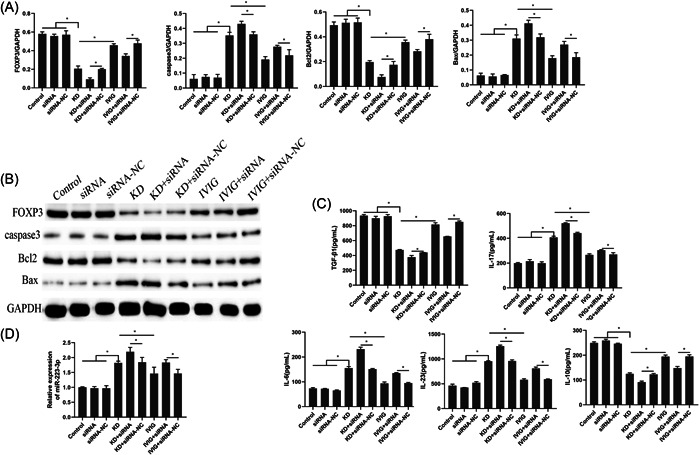
The effect of FOXP3 interference on the level of inflammation in cells. (A and B) The protein expressions of FOXP3, caspase3, Bcl2, and Bax. (C) The expression of inflammatory cytokines IL‐6, IL‐17, IL‐23, IL‐10, and TGF‐β1 was detected by ELISA. (D) The expression of miR‐223‐3p.

Compared with the control group, siRNA group and siRNA‐NC group, the expressions of FOXP3 and Bcl2 in the KD group were significantly downregulated, and the expressions of caspase3 and Bax were significantly upregulated. Compared with the KD + siRNA‐NC group, the results of the KD + siRNA group were consistent with the above. Compared with KD group, the expressions of FOXP3 and Bcl2 in IVIG group were significantly upregulated, and the expressions of caspase3 and Bax were significantly downregulated. Compared with IVIG + siRNA‐NC group, the expressions of FOXP3 and Bcl2 in IVIG + siRNA group were significantly downregulated, and the protein expressions of caspase3 and Bax were significantly upregulated, Figure 4A,B. At the same time, KD serum decreased the levels of TGF‐β1 and IL‐10, increased the levels of IL‐17, IL‐6, and IL‐23 (Figure 4C). The above results were more obvious when KD serum and siRNA were intervened together. In contrast, IVIG‐treated serum could raise the levels of TGF‐β1 and IL‐10 and decrease the levels of IL‐17, IL‐6, and IL‐23. In addition, miR‐223‐3p was significantly increased in the cells of the KD group compared to the control group. Compared with the KD + siRNA‐NC group, the KD+siRNA group also significantly increased miR‐223‐3p. The expression of miR‐223‐3p in cells was decreased in serum after IVIG treatment. These results indicated that miR‐223‐3p in the serum of KD patients may affect the apoptosis and inflammation levels of HCAECs by regulating the expression of FOXP3.

## 4 DISCUSSION

KD is a frequent febrile multisystemic inflammatory disease in children. A sequence of infectious cellular events, both innate and adaptive immune cells and platelet activation, occur in the blood of KD patients. Its early diagnosis is critical for effective treatment, which including IVIG and, for those who do not respond, additional IVIG or other anti‐inflammatory agents, eliminating the inflammatory process and reducing the risk of coronary artery aneurysm (CAA) to approximately 5%–10%.^16^ Impaired recovery from long‐term inflammatory damage leads to abnormal remodeling and severe coronary lesions in KD patients, according to previous studies.^17^ Hyperactive platelets in KD result from inflammation and endothelial damage, and platelet hyperactivation increases the risk of coronary syndrome.^18^ This study found that miR‐27b could target Smad7 and suppress the growth and immigration of HUVECs.^19^ KD serum can increase the level of miR‐186 in HUVECs and induce apoptosis in HUVECs by targeting SMAD family member 6 (SMAD6) to activate mitogen‐activated protein kinase (MAPK), which may become a therapeutic target for KD.^20^ Therefore, mining miRNAs related to HUVECs apoptosis is a direction of KD targeted therapy. It found that the level of miR‐223 was increased during LCWE‐induced KD vasculitis, but miR‐223 appeared to reduce inflammation in vascular tissue by inhibiting NLRP3 activation and IL‐1β production.^21^ It has also been found that the co‐culture of activated platelets and endothelial cells in KD patients will lead to the upregulation of miR‐223 expression in endothelial cells, and the upregulated miR‐223 will reduce the adhesion of leukocytes to endothelial cells.^22^ In another study, it was found that in KD patients, blood cell‐derived miR‐223 in vascular endothelial cells was elevated, and the increased miR‐223 may be involved in the vascular injury of KD as a new endocrine genetic signal.^13^ Therefore, there are currently inconsistent reports on the role of miR‐223 on endothelial cells. Consistent with our results, the expression of miR‐223 was increased in KD patients. The effect of miR‐223 on endothelial cells may be inconsistent due to the different research methods. Our results found that the expression of miR‐223‐3p was significantly upregulated in serum of KD during acute phase. Inflammatory blood cells derived from bone marrow hematopoietic cells can secrete miR‐223 into circulating serum, and miR‐223 secreted by blood cells can act as a new endocrine signal to enter vascular smooth muscle cells (VSMCs) and regulate its function and atherosclerosis through its target gene.^23^

Studies have shown that the proportion of Th17 (helper type 17) cells in the acute phase of KD is higher, and the expression of cytokines IL‐17, IL‐6, IL‐23 is increased, and the study also found that the proportion of Th17 cells in IVIG‐resistant patients was higher than that in IVIG response patient.^24^ These studies illustrate the pathological importance of T cells in children with acute KD. In this study, it was found that the proportion of Th17 cells in the serum of KD patients with acute was significantly increased, and the proportion of Treg cells was significantly decreased. Traditional Treg cells produce anti‐inflammatory cytokines such as IL‐10, transforming growth factor TGF‐β and IL‐35. The levels of IL‐6, TNF‐α, and IFN‐γ were significantly increased in the acute phase of KD, whereas the levels of IL‐6 and IFN‐γ decreased after IVIG treatment.^25^ In this study, the levels of IL‐6, IL‐17, and IL‐23 were increased, and the levels of IL‐10, TGF‐β1, and FOXP3 were decreased in patients with acute KD. The opposite results were obtained in the serum of patients after IVIG treatment. FOXP3 exerts immunomodulatory effects through various mechanisms including secretion of IL‐10 and TGF‐β.^26^ Through clinical data, it was found that the expression of FOXP3 was downregulated in the serum of acute patients. At the same time, the expression of FOXP3 was also downregulated in HCAECs cells containing KD serum. In addition, overexpression of miR‐223‐3p was also found to promote cell apoptosis. This is consistent with the result that highly expressed miR‐223‐3p in KD serum promotes apoptosis. miR‐223‐3p may regulate the apoptosis of coronary endothelial cells by regulating FOXP3, and then participate in the progression of KD.

Inflammatory cytokines are released by a variety of cells in KD. These inflammatory factors are attributed to the activation of apoptosis.^27^ In addition, KD involves the activation of innate and adaptive immunity, leading to the release of cytokines and the creation of an inflammatory environment. Studies have found that cytokines released by THP1 play an important role in acute KD vascular endothelial injury, THP1 cell line has been widely used as an in vitro model of human monocytes and macrophages for the study of the mechanism of inflammatory diseases.^28^, ^29^, ^30^ The THP1 cell line is essential for inducing inflammatory conditions in KD. We established a co‐culture system between HCAECs cells transfected with FOXP3 siRNA and THP1 cells added with three serum. The results showed that both high expression of miR‐223‐3p and low expression of FOXP3 in KD serum increased cell apoptosis. Meanwhile, the protein expressions of caspase3 and Bax were significantly upregulated. In addition, the highly expressed miR‐223‐3p in KD serum decreased the level of FOXP3. It is suggested that miR‐223‐3p may play a role in KD by regulating FOXP3.

## 5 CONCLUSION

In conclusion, this study collected serum samples from acute KD, after IVIG treatment, and healthy controls. It was verified that KD serum has high level of miR‐223‐3p and low level of FOXP3. A co‐culture system was established with HCAECs cells and THP1 cells treated with three serum samples. It was verified that serum miR‐223‐3p in KD patients may affect cell proliferation, apoptosis, and the expression of inflammatory cytokines by regulating FOXP3.

## AUTHOR CONTRIBUTIONS

**Ronghao Zheng**: Review and editing (equal). **Jing Xie**: Review and editing (equal). **Weijie Li**: Formal analysis. **Jianping Shang**: Formal analysis. **Zuliang Shi**: Software. **Songbai Zhu**: Software. **Lin Gui**: Methodology. **Li Huang**: Methodology. **Lan Shu**: Conceptualization. **Donglei Liu**: Conceptualization. **Xiaofen Huang**: Conceptualization. **Yi Gon**g: Review and editing. **Xiaohui Li**: Review and editing. **Wanxia Chai**: Review and editing. **Xiaolin Wu**: Writing—original draft (lead). **Jing Yue**: Writing—original draft (lead).

## CONFLICT OF INTEREST STATEMENT

The authors declare no conflict of interest.

## ETHICS STATEMENT

This study was obtained permission from the Medical Ethics Committee of Hubei Provincial Maternal and Child Health Hospital (2020IECXM029). Before participating in this study, notified consent was obtained from each patient.

## Data Availability

The data used to support the findings of this study are included within the article.
